# Motility Enhancement through Surface Modification Is Sufficient for Cyanobacterial Community Organization during Phototaxis

**DOI:** 10.1371/journal.pcbi.1003205

**Published:** 2013-09-05

**Authors:** Tristan Ursell, Rosanna Man Wah Chau, Susanne Wisen, Devaki Bhaya, Kerwyn Casey Huang

**Affiliations:** 1 Department of Bioengineering, Stanford University, Stanford, California, United States of America; 2 Carnegie Institution for Science, Department of Plant Biology, Stanford University, Stanford, California, United States of America; 3 Department of Microbiology and Immunology, Stanford University School of Medicine, Stanford, California, United States of America; North Carolina State University, United States of America

## Abstract

The emergent behaviors of communities of genotypically identical cells cannot be easily predicted from the behaviors of individual cells. In many cases, it is thought that direct cell-cell communication plays a critical role in the transition from individual to community behaviors. In the unicellular photosynthetic cyanobacterium *Synechocystis* sp. PCC 6803, individual cells exhibit light-directed motility (“phototaxis”) over surfaces, resulting in the emergence of dynamic spatial organization of multicellular communities. To probe this striking community behavior, we carried out time-lapse video microscopy coupled with quantitative analysis of single-cell dynamics under varying light conditions. These analyses suggest that cells secrete an extracellular substance that modifies the physical properties of the substrate, leading to enhanced motility and the ability for groups of cells to passively guide one another. We developed a biophysical model that demonstrates that this form of indirect, surface-based communication is sufficient to create distinct motile groups whose shape, velocity, and dynamics qualitatively match our experimental observations, even in the absence of direct cellular interactions or changes in single-cell behavior. Our computational analysis of the predicted community behavior, across a matrix of cellular concentrations and light biases, demonstrates that spatial patterning follows robust scaling laws and provides a useful resource for the generation of testable hypotheses regarding phototactic behavior. In addition, we predict that degradation of the surface modification may account for the secondary patterns occasionally observed after the initial formation of a community structure. Taken together, our modeling and experiments provide a framework to show that the emergent spatial organization of phototactic communities requires modification of the substrate, and this form of surface-based communication could provide insight into the behavior of a wide array of biological communities.

## Author Summary

Communities of bacterial cells exhibit social behaviors that single cells cannot engage in alone. These behaviors are often a product of direct interactions that allow cells to communicate with each other. In the unicellular photosynthetic cyanobacterium *Synechocystis*, groups of cells collectively move along a surface toward a light source in characteristic, spatial patterns that do not seem to require that cells directly communicate or change their individual behavior. By varying the direction of the light source, we show experimentally that cells indirectly interact by secreting a substance that allows them to move more rapidly and to follow the paths left by other cells. We develop a biophysical model demonstrating that this form of interaction is sufficient to reproduce our experimental observations, and complement this model with simulations and physical scaling laws that provide a useful tool to design and interpret future experiments. Based on our results, we propose that physical modification of the cellular microenvironment may play an important role in inducing group behaviors in other systems.

## Introduction

The collective migration and spatial organization of cellular communities are often the result of integration of chemical signals [1], [2], spatial cues, and epigenetic differentiation within the population [3]. In natural environments, microbes live in communities that range from relatively simple to very complex in terms of species diversity [4]–[10], structure [11]–[13], and metabolic functions and pathways [14]–[16]. In complex communities, such as medically relevant biofilms [17], [18], swarms of social bacteria such as *Myxococcus xanthus* [19], [20], or microbial mats in the environment [4]–[6], community structure can be dynamic, involving the collective migration of cells in response to environmental cues [21], [22], and may depend on the production of an extracellular matrix, which can facilitate stabilization of spatial structure [23]–[27]. In the face of this complexity, mechanistic models of cellular interactions that recapitulate environmentally relevant community behaviors can enhance our understanding of structure-function relationships, particularly those that are at the interface of biological and physical phenomena. Since many of these interactions are dynamic and not easily amenable to standard genetic and molecular analysis, biophysical models built on experimental observations have the potential to make testable predictions by connecting cellular behaviors to community-scale architectures.

One such example of community behavior is the directed surface-dependent motility of cyanobacteria either toward or away from a light source [28], [29]. This phenomenon, known as phototaxis, is easily visualized in the unicellular cyanobacterium *Synechocystis* sp. PCC 6803 (hereafter *Synechocystis*). In a typical phototaxis assay, cells spotted on a wet surface such as a low concentration agarose plate and placed in a directional light source begin to move in coordinated groups, followed by the formation of finger-like projections [30], [31]. To explore the molecular underpinnings of this striking community behavior we have previously used a combination of forward and reverse genetics [32]–[34]. These approaches have revealed that motility requires Type IV pili (TFP). TFP are multifunctional appendages found in many bacterial phyla and are required for surface-dependent motility, adhesion, and competence [35]–[38]. In addition, a number of photoreceptors [28], [39], [40], surface proteins [41], and signaling molecules such as cyclic AMP [41] appear to be involved in this highly regulated behavior. However, it remains unclear how single cells with a limited light bias [42] eventually organize into large groups of cells that exhibit predictable, coordinated phototactic behavior.

To dissect this community behavior, we developed a minimal biophysical reaction-diffusion model based on our experimental observations in which cells undergo a light-biased random walk with motility dictated by the local concentration of a cell-secreted substance. Simulations based on this model recapitulate the wide range of observed motility patterns. Furthermore, exploration of the phase space of this model showed that varying the cell density, light bias, and persistence of the cell-dependent surface modification could tune the shape, dynamics, and steady-state speed of the community, consistent with experimental observations. Based on physical arguments and our computational modeling, we present heuristics for the scaling of these features that could apply to a broad class of motile, structured communities. We were also able to confirm key qualitative predictions of our model by performing experiments in which we systematically varied the initial cellular concentration of the community. Thus, the computational models developed in this study predict that the physical properties of cellular microenvironments play a critical role in regulating single-cell behavior and that these behaviors are transduced into community organization.

## Results

### Cyanobacterial motility is coupled to surface modification

**Figure 1:**
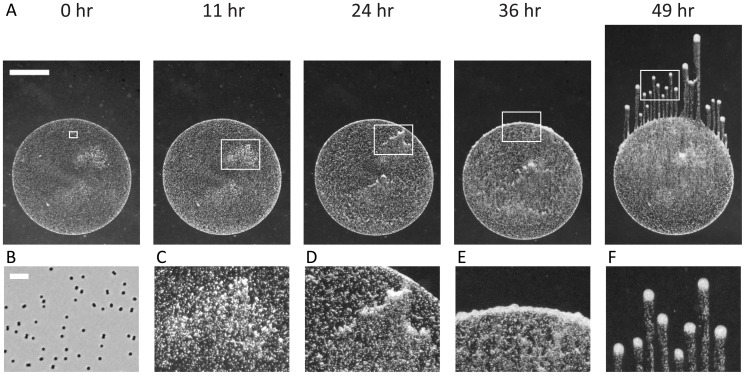
*Synechocystis* cells on a surface accumulate in finger-like projections when moving toward a directional light source. A) Time-lapse imaging of a *Synechocystis* community in the presence of a directional light source; images were taken at 0, 11, 24, 36, and 49 hr. White rectangles indicate regions highlighted in (B–F). Scale bar is 1 mm. B) Cells are initially randomly distributed across the surface. (phase contrast image taken at 10× magnification, scale bar is 10 µm.) C) Regions of heterogeneity are evident where the local cell concentrations are slightly higher than in the rest of the initial deposition area. D) Regions of higher cell concentrations start to form small finger-like projections within the initial deposition. E) Cells collect at the edge of the initial deposition and create a ruffled interface, from which finger-like projections will emerge. F) Finger-like projections form as distinct, motile groups of cells that move toward the light source, leaving behind a trail of lower density cells.

We used a well-established phototaxis assay in which a small volume of exponentially growing *Synechocystis* cells was spotted onto a low-concentration (0.4%) agarose plate, which was subsequently placed in the path of a directional light-emitting diode (LED) light source and imaged using time-lapse microscopy (Materials and Methods) [41], [42]. Typically, cells were initially randomly distributed across the surface and exhibited motility within 30 minutes after spotting. Within a 12–24 hour period many cells had migrated to the edge of the spot closest to the light, resulting in a typical crescent-shaped grouping of cells; next, a ruffled edge formed, indicating a transition in which cells begin to separate into spatially distinct groups. After 24 hours, long (mm-scale), finger-like projections were formed in which the majority of the cells accumulated at the tip and the group moved in a nearly straight-line path toward the light source (Fig. 1).

**Figure 2:**
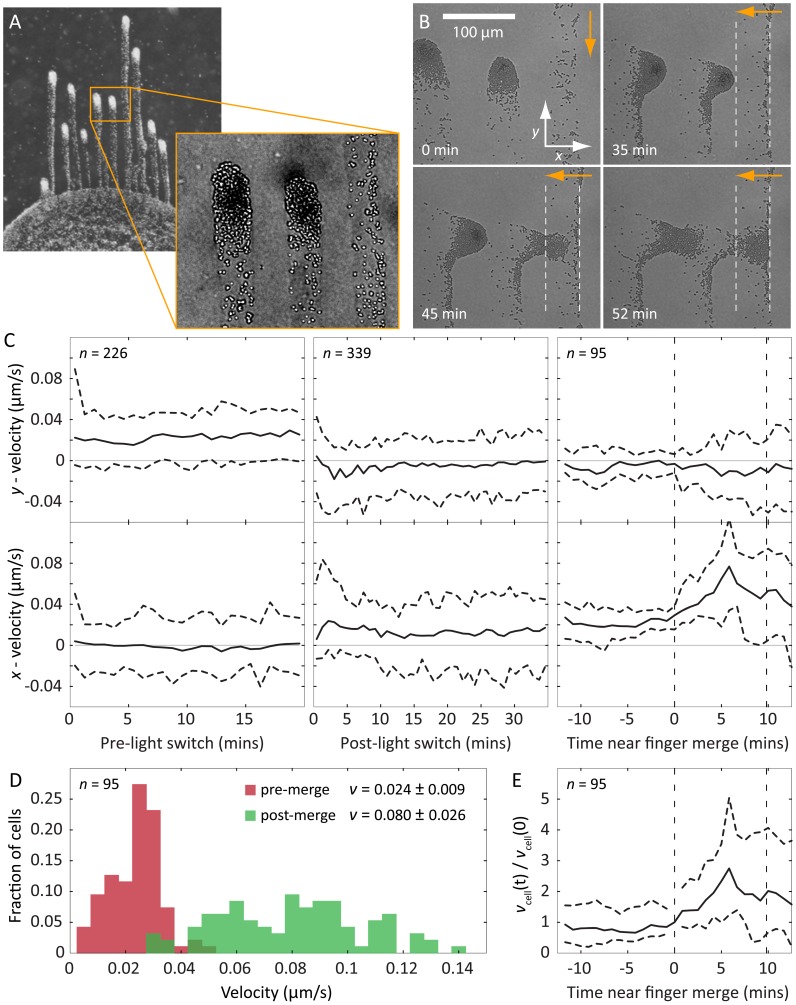
Cells secrete an extracellular substance that enhances their motility. A) Typical fingering of a *Synechocystis* community toward a light source during a phototaxis assay. The contrasted inset shows EPS deposited by motile groups. B) Phase-contrast microscopy of motile groups immediately before, and then at specified intervals after a change in the light direction (orange arrows). The white dashed lines indicate the bounds of a region through which a finger recently passed. The motile group changes course after a change in the light direction, and upon intersection with the EPS trail of a neighboring finger, group speed toward the light increases and the cells become more dispersed. C) Median velocity of cells in the motile group in both the *x* and *y* directions with respect to the coordinate system shown in (B); dashed horizontal lines indicate 95% confidence intervals. Left: prior to the change in light direction, cells have positive velocity toward the light source (top), and approximately zero net velocity perpendicular to the light (bottom). Middle: following the change in light direction, the cells reorient and velocity in the *x* direction rises to a value comparable with the *y* velocity prior to the light change (bottom), while the net y velocity approaches zero (top). Right: when the group of cells merges with the trail of the neighboring finger (dashed vertical lines), the spread in *y* velocities increases (top) and the median *x* velocity increases by approximately three-fold (bottom). D) Histograms of speeds for the same cells (*n* = 95) before and soon after merging with the trail of secreted extracellular substance, with mean speed and standard deviation indicated in the legend. E) Individual cells experience an increase in speed after the group merges with the trail of another finger, indicating that the change in group dynamics upon merging is coupled to a change in the motility of individual cells.

The spatially separated, finger-like projections were surrounded by an optical halo distinguished by a different index of refraction from the surface (Fig. 2A inset). Moreover, cells at the front of a moving finger left behind a trail that was subsequently followed by other cells. This suggested that the material in the trail might have specific properties that affect cellular motility. To test this hypothesis, we reoriented the light direction by rotating the plate 90 degrees. The tips of the fingers, where the cell concentration was highest, reoriented and moved toward the new direction of the light source within a few minutes after rotating the plate (Fig. 2B,C), indicating that the time scale of change in the direction of light bias was short compared to that of finger formation. Using custom tracking software to measure the instantaneous velocities of single cells in the fingertip (Materials and Methods), we determined that the cells re-established their previous steady-state velocity distribution within approximately 5 minutes after turning (Fig. 2C).

When the cells in one finger encountered the trail left by cells in a neighboring finger, we observed two changes that indicated that the trail affected motility. First, cells in the merging finger sped up upon encountering the trail left by a neighboring finger: both the mean and width of the velocity distribution increased approximately three-fold, indicating a faster and less coordinated group of cells (Fig. 2D). Second, the cells in the merging finger became more dispersed, indicating a reduction in the need for group coherence during movement. These observations indicate that trails left by cells locally enhance the motility of other cells, and groups of cells intersecting these trails can maintain their motility without maintaining the same levels of aggregation. Thus, our results suggest that cells secrete an extracellular substance that alters the agarose surface properties to increase motility. Although the composition and specific nature of this extracellular substance are unknown, we will refer to it as extracellular polymeric substance (EPS), by analogy with other community-forming species [16], [43] such as *Myxococcus xanthus* in which secreted substances play an important role in motility and group behaviors [44]. These observations of cell-mediated surface modification motivated the development of a biophysical model that could reveal the minimal requirements for finger formation.

### A minimal biophysical model that reproduces observed community dynamics during phototaxis

**Figure 3:**
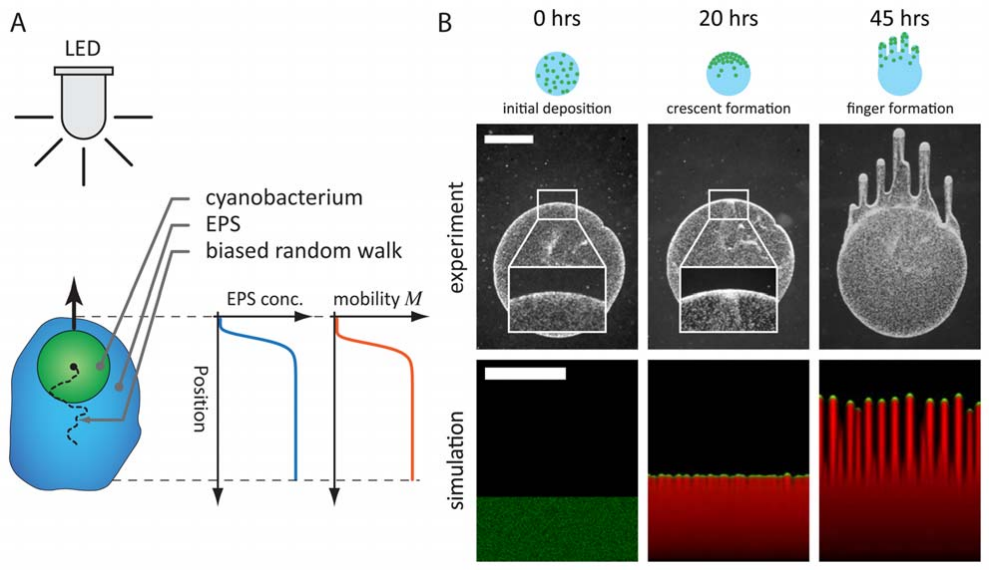
Biophysical model of EPS-based mobility enhancement captures the observed fingering behavior during phototaxis. A) As shown schematically, each cyanobacterium (green) is assumed to undergo a biased random walk toward the light source (LED). Each cell secretes EPS (blue), whose local concentration increases the cell's mobility *M* (red). B) The cell deposition, crescent formation, and finger formation observed in experiments (top) are recapitulated by simulations using our model (bottom) with 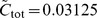 and 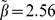; cells are shown in green, EPS in red. Immediately after deposition (left), cells are distributed randomly and the boundary with the substrate is smooth (inset). Cells collect at the edge of the initial deposition area as they migrate toward the light source (middle, 20 hr), with small variations at the interface (inset). The front matures into discrete groups of cells that separately migrate toward the light source (right, 45 hr). Scale bars are 1 mm.

Our finger merging experiments indicated that the motility and coherence of cellular groups at the tips of *Synechocystis* fingers change on the time scale of minutes once cells encounter a pre-existing EPS trail, suggesting that cell movement is dependent on the local EPS concentration (Fig. 3A). In contrast to previous models that rely on direct cell-cell communication or variable single-cell behavior to produce fingering patterns [30], [31], [45], we hypothesized that the observed spatiotemporal community dynamics could be explained simply by cell-mediated physical alteration of the surface (Fig. 3B). To test this prediction, we developed a reaction-diffusion model linking cellular and EPS concentrations to motility and the motion bias due to a directed light source.

We assume that cells produce EPS at a constant rate *k_s_* such that
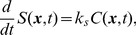
(1)where 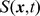 is the EPS concentration at a point ***x*** on the two-dimensional surface at time *t*, and 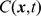 is the cellular concentration. Both concentrations are measured as the height that the EPS or cells would occupy if locally spread with a uniform thickness. In the absence of a directed light source, previous single-cell tracking experiments revealed that cells move in an approximately random walk fashion [42]. To incorporate our observations of EPS-mediated motility, we defined a phenomenological function *M* that is an effective diffusion constant that can vary in space and time; we assume that its spatiotemporal dependence is incorporated through the EPS concentration 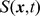. By analogy with physics nomenclature, we term 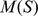 the cellular mobility, since it describes the ease with which cells move across a surface. Based on our experimental observations that cells exhibit increased movement at higher EPS levels, the mobility should be a monotonically increasing function of the EPS concentration, and we assume the mobility saturates at high levels of EPS. To limit the number of parameters in this function, we assume a simple functional form
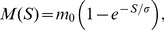
(2)where *σ* is the saturation depth above which additional EPS does not significantly increase the mobility, and *m*_0_ is the maximum mobility in the presence of saturating EPS.

In the absence of a directed light source, we assume based on the observed, approximately random walk behavior that the cellular concentration spreads diffusively over time [42]. The effect of light is to bias the random walk toward the light source, and thus we model the cellular flux ***J*** as
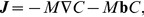
(3)where the first term on the right hand side corresponds to the flux from diffusive random motion of the cells, and the second term corresponds to the flux driven by a force vector **b** pointed toward the light source whose magnitude corresponds to the strength of the light bias. Since the distance to the light source is much larger than the cellular community even after fingering, we assume that **b** has constant magnitude and direction throughout the typical time scale of an experiment, except in simulations designed to mimic our finger merging experiments. The flux from Eq. 3 determines the dynamics of the local cellular concentration through
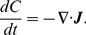
(4)


For simplicity, we ignore reproduction in all of the following simulations in order to define the phase space of community patterns in terms of a fixed total cellular mass 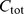; we note that our model can easily be modified to account for nonzero rates of reproduction. For a light source incident from the *y* direction, with corresponding net motion also in the *y* direction and spatially varying mobility *M*, Eq. 4 becomes the biased diffusion equation
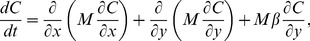
(5)with nonlinear behavior emerging from feedback to the local EPS production rate through the spatially varying mobility *M*. With these assumptions, our model has four parameters: *k_s_*, *m*_0_, *σ*, and the magnitude of the constant bias vector, 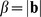. However, dimensional reduction reduces the number of free parameters without any loss in descriptive power. We define a natural time scale by 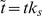, a natural length scale by 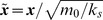, and a natural density scale for cell mass and EPS by 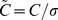 and 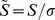. The model is therefore reduced to two dimensionless parameters: (i) the mean initial cellular concentration, 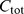, normalized by the mobility saturation concentration *σ*, and (ii) the bias strength, *β*, normalized by 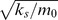. The model then takes a simpler form where variables marked with a tilde are understood to be dimensionless. The EPS concentration evolves according to
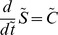
(6)and relates to the mobility by 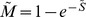, where 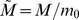 is the normalized mobility. The cellular concentration field evolves according to
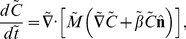
(7)with 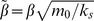
and the spatial derivatives normalized by 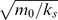. This reduction of the parameter space to two free variables, 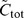 and 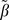, dramatically simplifies the comprehensive mapping of system behavior without any loss of information or generality.

**Figure 4:**
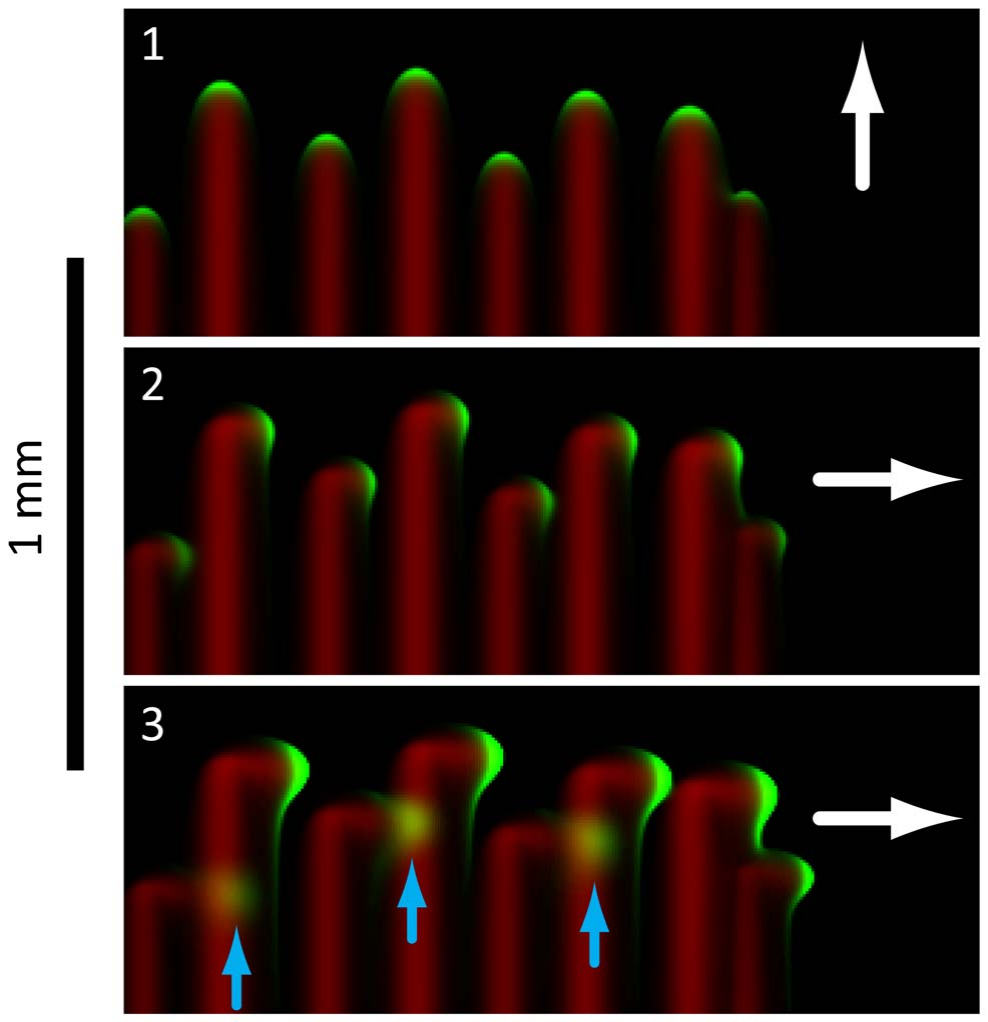
Biophysical model reproduces key features of group motility during finger merging. In these simulations, the direction of light bias (white arrows) is rotated 90 degrees between frames 1 and 2. The cells (green) leave an EPS trail (red), and when a finger intersects the EPS trail left by a neighboring finger, the group of cells speeds up and spreads out (blue arrows, frame 3). We observed the same qualitative changes in finger merging experiments (Fig. 2).

In order to determine the conditions under which our model predicts phototactic fingering to occur and the types of spatiotemporal dynamics that are accessible, we explored the phase space of emergent behaviors by solving our reaction-diffusion model (Eqs. 6 and 7) numerically for a wide range of values of 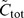 and 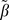. Although the equations are deterministic, we introduce stochasticity by initiating each simulation with a random distribution of cells over a fixed, contiguous portion of the simulation area with a given mean value 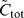. Each simulation started with zero initial EPS concentration. To mimic a directed light source in the far field, a constant light bias was oriented toward the top of the rectangular simulation area (Fig. 3A). For a moderate value of the mean cellular concentration 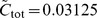 and a bias force 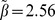, our simulations recapitulated the initial gathering of cells at the front of a spot and the subsequent ruffled edge, and eventually developed distinct, finger-like projections similar to those observed in experiments (Fig. 3B). To link our dimensionless parameters to the length and time scales exhibited by our experiments, we obtained estimates of the microscopic parameters 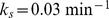 and 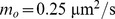 from the expansion of the EPS halo and single-cell movements, respectively (Materials and Methods). This predicted a time scale for fingering of approximately 24 hours, in agreement with our experiments. In addition, the natural length scale 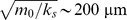, which defines the distance over which motion due to cellular diffusion is limited by the rate of EPS secretion, was in agreement with the characteristic widths of fingers in our experiments. Finally, our model reproduced the dynamic changes that we observed experimentally when one motile group encounters the EPS trail of a neighboring motile group (Fig. 4); the concentration field of the incident motile group exhibited both the rapid increase in speed and group de-coherence (Fig. 2B).

**Figure 5:**
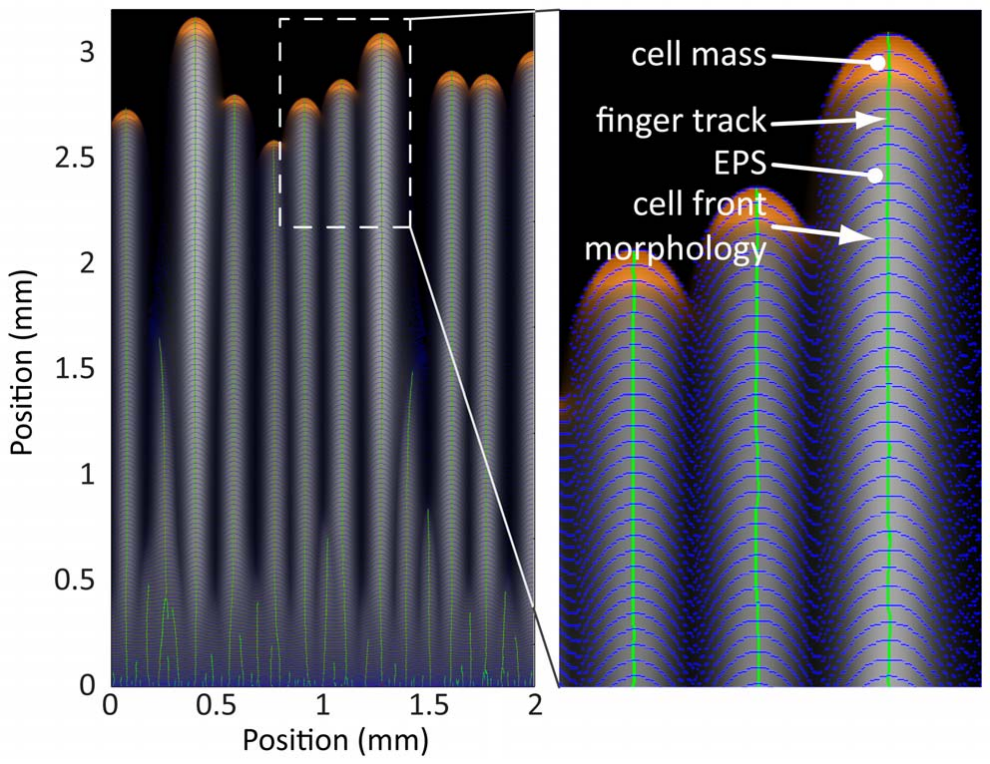
Quantitative analysis of community morphologies from a representative simulation. Using computational image processing at each time point, we quantified the number of fingers and their ramp time, size, and speed. The inset shows the cells (orange), cell front morphologies at evenly-spaced time points (blue contours), the group center positions over time (green lines), and the EPS field at the final time point (grayscale). These data provide a set of quantitative metrics for mapping model parameters to community behaviors.

These simulations of cellular and EPS concentrations exhibited morphological and dynamic properties that were amenable to quantification (Fig. 5). Simulated cell density images were computationally segmented to track the size (measured in units of volume), position, and velocity of each distinct motile group (finger) over time, and the corresponding EPS concentration field was used to track when two nearby fingers merged. We also measured the time scale associated with the transition from a random distribution of cells to steady-state motion of motile groups toward the light source, which we refer to as the “ramp time.” This comprehensive exploration allows us to map the phase space of possible behaviors, and determine scaling laws linking features such as finger speed to mean cellular concentration that provide insight into the physical consequences of motility feedback via surface modification.

### Phase diagram of community morphologies and behaviors

**Figure 6:**
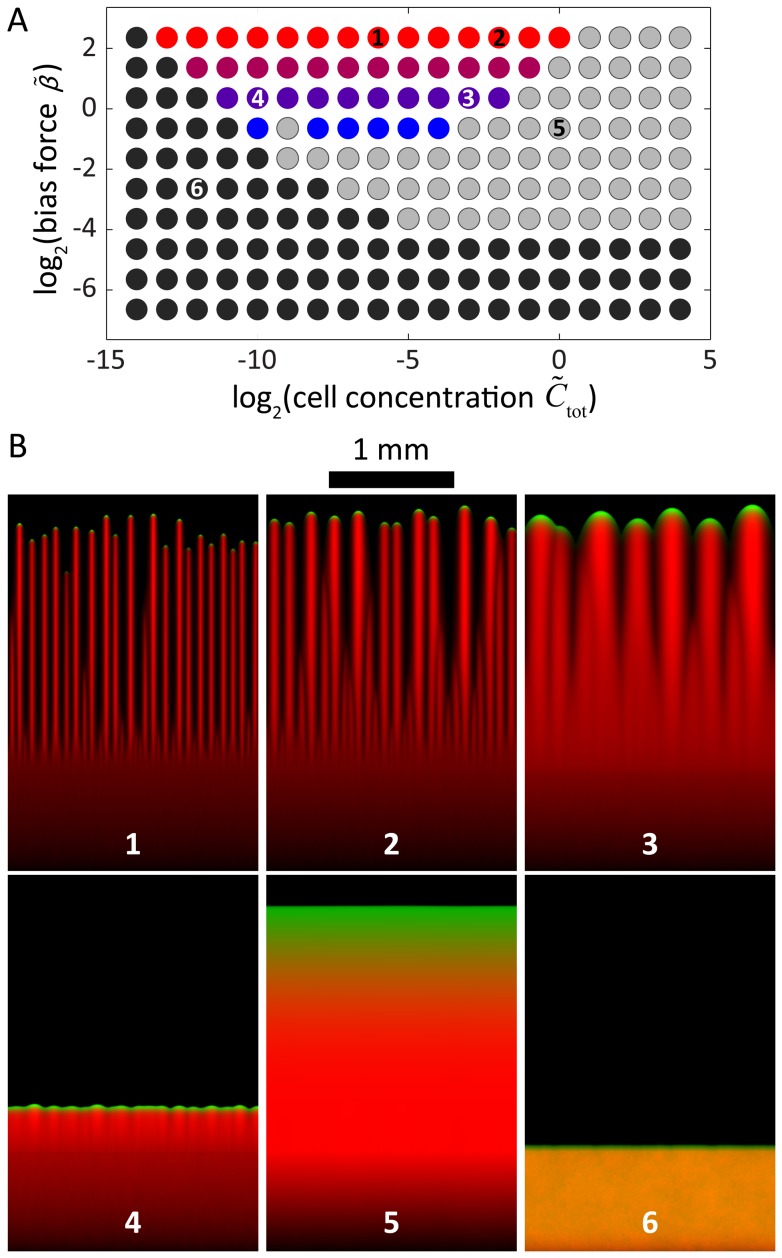
Computational determination of the phase space of collective motility behaviors. A) Simulations grouped into classes as a function of dimensionless bias force and mean cellular concentration, resulting in the phase diagram shown on logarithmic axes. Colored circles represent parameters with a sufficient degree of directed movement and cell front instability to result in simulations with finger-like morphologies. Different shades correspond to the values of the bias force. In the simulations represented by the grey circles, cells move in a directed fashion toward the light source but do not spontaneously gather into groups due to high cell density, and thus high local EPS production. For the simulations represented by the black circles, little to no cellular movement resulted due to insufficient EPS production and/or bias force. Numbers correspond to the frames in (B). B) Representative morphologies at the end of simulations with parameters from different regions of the phase diagram. In numbered frames, cells are shown in green, EPS is shown in red, and orange indicates co-localization of cells and EPS. The simulations in frames 1–4 have finger-like morphologies with varying group sizes and spacing. The simulation depicted in frame 5 has a moving but uniform cell front (i.e., no distinct groups), while frame 6 reflects a simulation of essentially non-motile cells.

To quantify the extent of the region of parameter space for which our model produces morphologies relevant to the biological system, we used simulations to comprehensively map the space of possible community behaviors by varying the dimensionless total cellular biomass and the dimensionless light bias strength (Fig. 6A; Materials and Methods). Within the range of parameters studied, a region emerged in which our model generated motile groups of varying sizes and speeds, with a wide range of time scales for the establishment of steady state motion. In addition, large subsets of simulations with other parameter values exhibited qualitatively different morphologies from the characteristic finger-like projections typically seen in experiments (Fig. 6B).

Given the complex range of behaviors represented in our simulations, we wrote custom software to quantify the community morphologies at every simulation time point (Materials and Methods). Simulations were split into three classes according to the overall degree of cellular movement and the magnitude of non-uniformity of the advancing front, forming a phase diagram with dimensionless cellular concentration and bias force as the independent parameters (Fig. 6A). For every pair of values 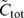 and 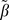, we performed three simulations with different initial random distributions of cells. For all cases, the classification of resulting morphologies was consistent across initial conditions; finger speed and ramp time typically varied by only ∼5% and ∼10%, respectively. In the first class (colored dots in Fig. 6A), cells had sufficient EPS production to become motile, and sufficient bias force to generate finger-like projections; the number and size of the motile groups varied depending on the parameters (Fig. 6B, frames 1–4). In the second class (gray dots in Fig. 6A; Fig. 6B, frame 5), the community was motile, but the high concentration of cells led to high levels of EPS production, causing the front to advance uniformly without splitting into distinct groups. In the third class (black dots in Fig. 6A; Fig. 6B, frame 6), the community was non-motile due to a relatively weak light bias and/or a low cellular concentration that was insufficient to produce enough EPS for movement over the time scale of the simulation. Therefore, our simulations predict that for a range of cellular concentrations and light biases outside the finger formation region, cells should exhibit uniform and/or non-motile fronts.

### Scaling of group size, speed, and ramp time with cellular concentration

**Figure 7:**
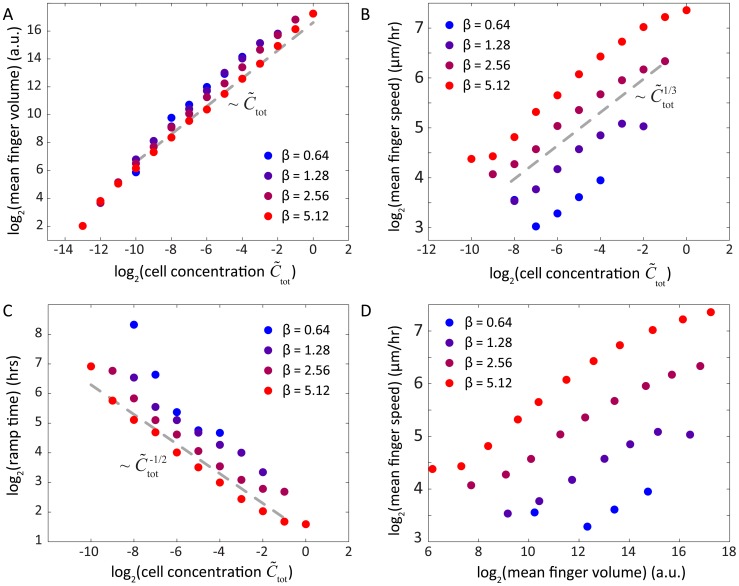
Scaling laws reveal simple relationships among cell concentration, light bias, and community morphology. A) The number of cells in each motile group as a function of initial cellular concentration follows an approximately linear relationship independent of bias force (gray dashed line). B) The mean speed of cellular groups approximately follows a 1/3-power law. C) The ramp time associated with the formation of finger-like morphologies follows an approximately inverse square-root power law. D) The number of cells in each finger and the speed toward the light source are positively correlated for all bias forces. In all panels, colors refer to the bias force legend and correspond to dots in the phase diagram of Fig. 6A.

For the subset of parameters that exhibited finger-like projections, we quantified motile group size, speed, and ramp time as a function of mean cellular concentration (Fig. 7) to extract general rules that underlie community behavior. In addition, we used physical arguments to predict the scaling properties of each of these variables that could be compared with our numerical simulations. Based on our measurements of *m*_0_ and *k_s_*, the natural length scale, 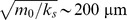, sets the approximate width of a finger-like projection, independent of the cellular concentration or bias force. In our simulations mimicking a 2 mm wide region of the surface, we expected approximately 10 distinct finger-like projections, with some amount of random variation. Indeed, over the relevant region of phase space in Fig. 6A, the number of distinct motile groups ranged from 5–20, with only a slight dependence on mean cellular concentration, which varied by more than three orders of magnitude in the simulations, and bias force, which varied by an order of magnitude. Thus, by linking cellular-scale properties (*m*_0_ and *k_s_*) to the patterning of community-scale motility, this physical argument successfully predicts that the number of fingers should remain relatively constant as the mean cellular concentration increases, while the number of cells in each finger increases approximately linearly (Fig. 7A).

In our simulations, each finger reaches a steady-state velocity that approximately scales as the mean cellular concentration to the 1/3 power (Fig. 7B). This scaling can also be explained via the relationship between EPS production and cellular concentration. The rate at which a group of cells secretes EPS is proportional to the size of the group (Eq. 6). At steady state, this rate is balanced by the rate at which the front of cells in each group deposits EPS onto virgin substrate during the forward motion of the finger, which is proportional to (i) the depth of the EPS trail, (ii) the width of the trail as dictated by the finger width, and (iii) the forward velocity of the finger. If we assume that cells cluster at the tip of an individual finger in a shape that can be reasonably approximated by a hemisphere, then the width of the trail should scale as the number of cells to the 1/3 power. Taken together, the conservation of EPS secretion and deposition rates for a single finger at constant velocity dictates that
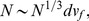
(8)where *N* is the number of cells in the finger, *d* is the depth of the EPS trail, and 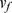 is the steady-state velocity of the finger. For the low values of the dimensionless mean cellular concentration (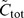) that produce finger-like projections, cellular mobility is linear in the depth of the EPS trail (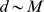). Likewise, the velocity of a finger moving under light bias is proportional to the mobility (Eq. 3; 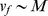), and therefore the depth of the EPS trail is proportional to the velocity (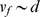). In combination with Eq. 8, this analysis gives 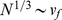; since 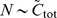 (Fig. 7A), the predicted scaling relationship is 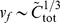, which is demonstrated empirically in Fig. 7B.

For the initial, transient phase of our simulations, when cells begin to aggregate into distinct fingers, the ramp time is the time scale over which the fingers reach a terminal velocity. In this phase, the bias force leads small collections of cells to move forward with an approximately constant velocity as they travel over the EPS left by cells in front of them. The cells eventually collect at the leading edge of the initial cellular deposition, a behavior that mimics the crescent morphology observed experimentally in Figs. 1 and 3B. The rate at which cells accumulate at the leading edge is proportional to the velocity of these small groups of cells and the mean cellular concentration, such that the concentration of cells at the leading edge increases as
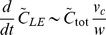
(9)where 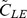 is the concentration at the leading edge, 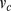 is the constant velocity of cells heading toward the leading edge, and *w* is the relatively fixed width of the crescent zone. Thus, the concentration of cells at the leading edge scales roughly as 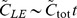, and hence the concentration of EPS at the leading edge 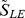 scales quadratically in time as 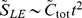. The transient phase ends when enough EPS has been deposited at the leading edge such that a finger achieves terminal velocity, and therefore the ramp time to reach a fixed level of EPS should scale as 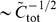. Our simulations predict a similar scaling (Fig. 7C), indicating that a higher initial concentration of cells will lead to the faster development of fingers.

**Figure 8:**
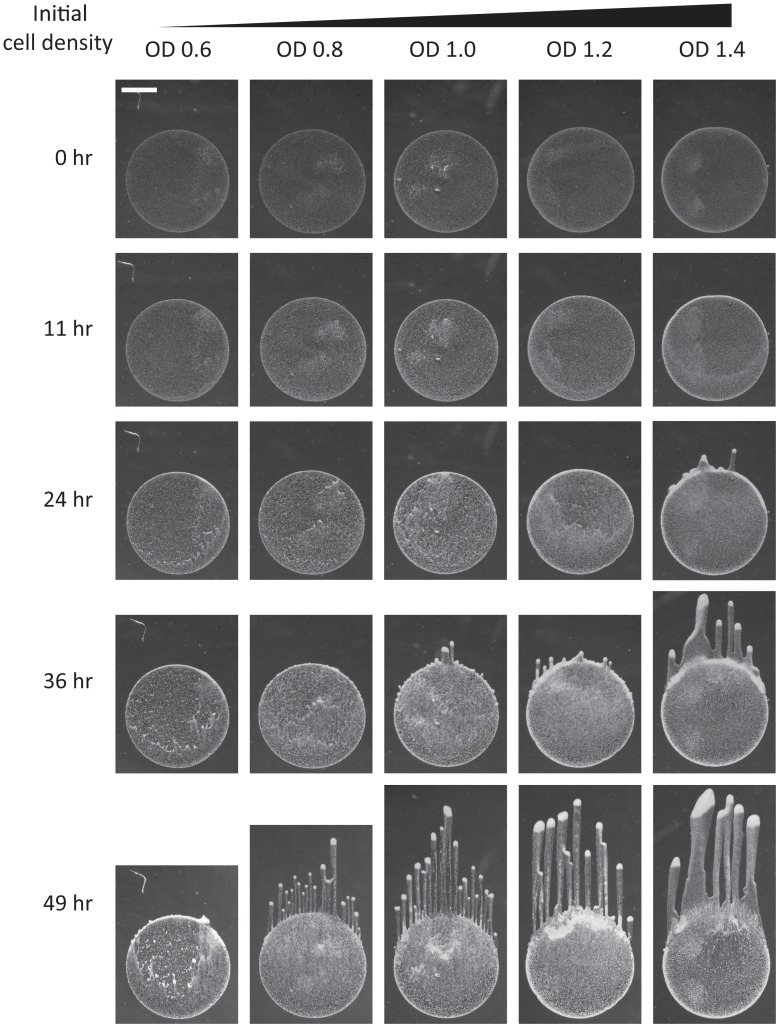
Model successfully predicts effects of increased cellular concentration of finger ramp time and biomass. Phototaxis time-lapse of *Synechocystis* cells deposited on an agarose surface with increasing initial concentrations with OD_730_ (from left to right) of 0.6, 0.8, 1.0, 1.2, and 1.4. Time-lapse images were taken at 0, 11, 24, 36, and 49 hr. The images show a negative correlation between initial cell density and time to finger formation, and a positive correlation between initial cell density and number of cells in each finger, both of which are trends predicted by our biophysical model. Scale bar is 1 mm.

These physical arguments indicate that the rate of finger development, number of cells in a finger, and finger speed are all positively related to mean cell concentration and to each other, at all relevant bias forces (Fig. 7D). Similarly, increasing light bias is correlated with decreased ramp times, increased finger speeds, and a slight reduction in motile group size. To test these predictions, we performed experiments in which we systematically varied the initial cellular concentration and measured the community morphologies over time. As predicted by our model, the ramp time decreased and the number of cells in each finger increased with increasing initial cellular concentration (Fig. 8).

### Effects of a time-dependent decrease in EPS efficacy

**Figure 9:**
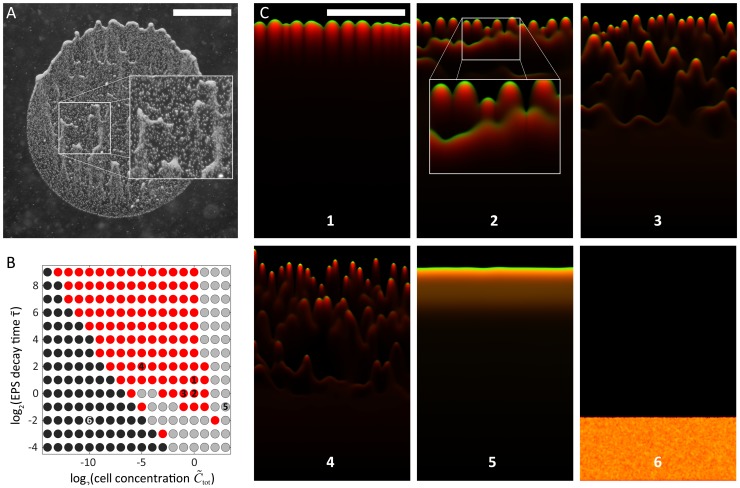
EPS decay results in multiple cell fronts and preferred paths for cellular groups. A) Experimental example of cells splitting into multiple fronts that head toward the light source. B) Phase diagram of the EPS decay model at a constant bias force. In the red region, cells form discrete, motile groups that head toward the light source in one or more cellular fronts. In the gray region, cells move toward the light source but do not form discrete groups. In the black region, cells are essentially non-motile due to insufficient EPS production and/or rapid decay of EPS. C) Late time-point snapshots of simulations from regions of the phase diagram in (B), with a light source at the top (green, cells; red, EPS; orange, colocalization of cells and EPS). Frames 1–4 depict results from simulations in the red region in (B), in which multiple fronts with finger-like morphologies can form similar to (A) with varying group sizes and numbers of distinct cell fronts. Groups of cells behind the primary front often catch up by following EPS trails left by forward groups (inset of frame 2). Frame 5 is from a simulation in the gray region of (B), in which cells move toward the light but do not form discrete groups. Frame 6 depicts the result from a simulation in the black region of (B); the cells are essentially non-motile. Scale bars are 1 mm.

While all experiments performed with sufficient initial cell number and in the presence of a directed light source showed the formation of distinct fingers at the front edge of the spot of cells, some experiments also displayed fingers forming within the deposition area (Fig. 9A, inset). We hypothesized that a time-dependent decrease in the efficacy of EPS-enhanced mobility, as would be the case if the EPS decayed or relied on a volatile component, contributed to this separation between the internal fingers and the front edge. To test whether this mechanism could produce multiple fronts, we introduced time-dependent EPS decay into our model by
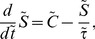
(10)where 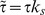 is a dimensionless time constant such that *S* decreases according to 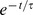 in the absence of cellular production of EPS.

For a given cellular concentration, the EPS concentration plateaus at a steady-state value 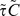. This limitation on the maximum value of the EPS concentration can cause a thresh-holding effect on the ability of a group of cells to move. If the relationship between mobility and EPS concentration, *M*(*S*), were switch-like (e.g. sigmoidal), cells in groups below a critical size would not be able to move because the steady-state EPS concentration would be too low. In contrast, in the absence of EPS decay, any group could move given enough time to produce sufficient levels of EPS. For the mobility function in Eq. 2, it is similarly the case that in the presence of EPS decay, the maximum mobility is no longer determined by how long a group of cells produces EPS at a particular location, but instead by how many cells are present in the group.

We performed simulations using our reaction-diffusion model with Eq. 6 modified to Eq. 10, with randomly distributed initial densities and the same motility relationship used for Figs. 4–7, at a fixed bias force of 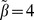 to ensure that the simulations encompassed a fingering region in Fig. 6A. We sampled values of the dimensionless time constant and mean cellular concentration to calculate a new phase diagram of system behaviors (Fig. 9B); these behaviors were divided into the same three classes depicted in Fig. 6A. For a wide range of cellular concentrations and EPS decay time constants, we found a region where cells split into distinct motile groups (red dots in Fig. 9B). Within that region, longer time constants resulted in slower decay of EPS that led to a single unstable front (Fig. 9C, frame 1), similar to Fig. 6B, frames 1–4. For shorter time constants, the initial mass of cells split into multiple non-uniform fronts, creating staggered motile groups (Fig. 9C, frames 2–4). We also identified a motile region without any fingering that resulted from high levels of EPS at higher cell concentrations (gray dots in Fig. 9B and Fig. 9C, frame 5). Finally, we identified a non-motile region caused by cell densities insufficient to sustain the EPS levels required for motility when decay times were short (black dots in Fig. 9B and Fig. 9C, frame 6).

Interestingly, in simulations that resulted in multiple fronts, motile groups that formed later and hence lagged behind the most forward groups often advanced by following the transient EPS trails of earlier fingers. Upon catching up, the two groups coalesced to form a larger, faster moving group that even more easily followed other transient EPS trails (Fig. 9C, inset of frame 2). This resemblance to our experimental data (Fig. 9A) suggests that the EPS trail can both separate cells into distinct groups (fingers) within a front, and gather separated fronts of cells into a single front via this dynamic coarsening mechanism.

## Discussion

We have developed a minimal biophysical model of the phototactic motility of *Synechocystis* cells whose behavior is regulated by cell density and the strength of the bias created by a directed light source. Our finger merging experiments, which indicated that modification of the agarose surface increases cell motility (Fig. 2), suggest that the two major factors underpinning fingering pattern formation are a positive bias towards light exhibited by single cells in combination with modification of the substrate surface. The close agreement between our model of *Synechocystis* motility and experimental observations of finger formation and subsequent reorganization after the plate was rotated relative to the light direction (Figs. 3, 4) suggest that phototactic fingering patterns are a consequence of how the physical properties of the surface, which are dynamically remodeled by the local population of cells, affect cell motility; no change in single cell behavior or direct cell-cell communication is required. The community swarming behavior of the bacterium *M. xanthus* has many similar features to *Synechocystis* phototaxis, including TFP-dependent motility and central role of EPS [20], [46], [47]. Agent-based models have shown that maximal outflow of cells from a *M. xanthus* swarm relies on regular reversals of EPS-mediated gliding [48] and a flexible, rod-like cell shape [49]. By contrast, *Synechocystis* phototactic patterns form independent of a known reversal mechanism, and cells are spherical in shape. Moreover, light provides a unique switchable cue with which to manipulate behavior, for example by rapidly altering finger trajectories (Fig. 2), making *Synechocystis* an excellent system for probing general properties of community motility.

Our model predicts the formation of distinct groups of cells (fingers) under a variety of light intensities (bias) and cell densities (Fig. 6). Finger volume is predicted to increase linearly with the cell density (Fig. 7A); in our simulations, larger fingers have a shorter ramp time (Fig. 7C) and collectively move more quickly toward the light with a speed that scales with the cell density (Fig. 7B). In contrast with extremely low cell densities, these results indicate that fingers can move in a more directed fashion toward light and at speeds that single cells cannot achieve. These conclusions are even more accentuated in simulations with increased rates of EPS decay, in which the motility of an individual cell toward a light source may be negligible in comparison with a group that can maintain a high local density of EPS (Fig. 9). Therefore, the model predicts that under a wide range of combinations of light bias, cell concentration, and EPS decay conditions, cells that are part of a finger are likely to exhibit increased motility [30]. Given that our analysis is based on a dimensionless set of reaction-diffusion equations, we note that the emergent behaviors predicted by our model are general properties of any system that uses surface enhancement and biased diffusion for motility. Several of these predictions corroborate what we have observed empirically in the laboratory. Our model now provides a rigorous framework in which various predictions can be further tested and refined using specific mutants or defined conditions. For example, our studies predict that EPS released into the medium by cell cultures or collected from the surface of the agarose may be provided exogenously to alter motility in spatially dependent patterns, and our computational model provides a useful tool for predicting the effects of such exogenous EPS addition.

Secreted EPS provides information about the concentration of cells that have recently resided at a particular location on the surface, a situation similar to chemical quorum sensing in which autoinducer molecules indicate the cell density of the population [50]. As *Synechocystis* groups develop, EPS trails provide a persistent mechanism of long-range, indirect communication that guides the coalescence of lagging groups with cells at the front of the drop. This dynamic coarsening of group size as cells move toward the light source is similar to water droplets on a window that follow the paths of previous droplets and coalesce to form larger water droplets that move even faster down their gravitational potential. Our model also suggests that EPS decay may play an important role in group motility by providing only a transient trail to guide other groups of cells that are farther from the light source (Fig. 9). While it may be advantageous to guide the groups immediately behind a leading group toward a light source, it is possible that changing environmental conditions such as light quality or direction may make it disadvantageous to have all cells exclusively follow the trail of cells at the leading edge.

Our goal is to connect the microscopic cellular properties incorporated into our model with the macroscopic, observable behavior of cellular communities. Whereas previous models explicitly assumed that neighboring cells experience local interactions or that cells switch between discrete states associated with different behaviors [30], [31], [45], our model seeks to reproduce the observed phototactic behavior using a minimal set of assumptions about the underlying factors controlling motility at the single-cell level. While our results do not rule out the possibility of chemical communication or changes in gene expression as contributors to the phototactic response of *Synechocystis*, our model provides a mechanistic explanation for finger formation that does not require these elements, and yet correctly predicts several trends in experimental data. In particular, we experimentally verified our model prediction that increasing the density of cells in the initial colony decreases the time required for finger formation and increases the subsequent finger size, with comparatively little variation in the number of fingers along the front of the spot (Figs. 7, 8).

Our use of a mean-field reaction-diffusion model assumes that the stochasticity of single-cell movement can be averaged over the population of cells; similar models have been applied to intracellular protein networks [51], [52] and have also been used to describe a combination of phototaxis and chemotaxis observed in *Dictyostelium* slugs [53], to model multiple competing cell populations [54], to explain phototaxis in non-equilibrium chemical systems [55], and to decipher the morphology of dendritic bacterial colonies [56], [57]. In each case, these models clarified the important factors for generating a particular behavior by demonstrating the sufficiency of a subset of potential variables. Although discrete, agent-based models can be used to study the behavior of a group of cells [42], [53], [58], we have focused on a continuous, mean-field model in order to map the behavior of an entire community across a large region of parameter space in a computationally tractable manner. It is relatively straightforward to introduce other factors into our model, including a diffusive chemical signal, cell-cell interactions mediated by Type-IV pili [35], crowding, or EPS production levels [59]. Future experiments examining single-cell behaviors will also help to elucidate whether these factors and/or stochasticity in single-cell motility are manifested. Moreover, quantitative characterization of community morphologies as a function of light intensity, direction, and wavelength should provide a calibration for the effective strength of the light bias in different conditions. Finally, targeted mutants in the synthesis of extracellular polysaccharides and varied surface properties will provide the opportunity to tune community dynamics and test predictions of our model.

The role of the local microenvironment in regulating both motility and the structure of the community can have a strong impact on a wide range of biological systems, including the migration of germ layer progenitor cells in the developing zebrafish embryo [60] and cancer cell metastasis [61]. The contribution of EPS to *Synechocystis* motility investigated in this study suggests that modification of surfaces as cells move across them may be an important parameter for understanding emergent community structure. The use of biophysical models to evaluate the physical basis of collective cell migration provides new avenues for further experiments and may underlie future efforts to control community behavior.

## Materials and Methods

### Growth conditions

*Synechocystis* sp. PCC 6803 cells were grown from an original single colony of phototaxis-positive cells in BG-11 media [62] at 30°C with continuous shaking at 100 rpm under overhead warm white fluorescent light (Super Saver Warm white F40WW/SS, 34W, Osram Sylvania Inc., MA, USA). All imaging experiments were performed using exponentially growing cells with OD_730_ = 0.6–1.3 (25,000–55,000 cells/µL; measured with an Ultrospec 3100 pro spectrophotometer, Amersham Biosciences, Sweden).

### Motility assay

Motility assays were carried out on 0.4% (w/v) agarose in BG-11 in 50-mm plastic petri dishes (BD Falcon, New Jersey, USA) at 30°C. One microliter of cells (OD_730_ = 0.8, or ∼40,000 cells) was placed in the center of a plate, and then inverted to minimize evaporation of the agarose. In Fig. 8, cells were diluted with fresh BG-11 media and one microliter of cells from each dilution was placed on a plate. A warm white LED (5 mm, 7000 mcd, 35° spread; Super Bright LEDs, MI, USA) was used to illuminate each plate. To induce directed phototaxis, the LED was placed 50 mm away from the center of the cell droplet, which was approximately 2.5 mm in diameter, at the level of the agarose. The incident light intensity was approximately 20 µmol photons/m^2^s, as measured with an LI-189 light meter (LI-COR Biosciences, NE, USA).

### Time-lapse imaging and cell tracking

Entire drops (Figs. 1, 2, 3, 8, and 9) were imaged using a Canon 60D DSLR camera (Canon U.S.A., Inc., New York, USA) attached to a Leica MZ12 stereoscope (Leica Microsystems, IL, USA). To induce cells to move into an existing EPS trail (Fig. 2), a Petri dish containing cells that had been under directional light for 24 hours was rotated by 90°. Time-lapse imaging at single-cell resolution was conducted at 20× magnification, 1 frame/sec, and 30°C using a Coolsnap-Pro Monochrome camera (Photometrics, Arizona, USA) attached to a Nikon TE-300 inverted microscope (Nikon Instruments Inc., Melville, NY, USA).

Cell tracking was performed using custom MATLAB (The Mathworks, Natick, MA, USA) software to quantify the positions and velocities of individual cells over time. In each frame, individual cells were segmented using thresh-holding and a watershed transform, and the locations of their centers of mass were recorded. The track of each cell was found using probabilistic nearest-neighbor connected-component analysis across frames. The average speeds of single cells were calculated from the total path length traveled over the preceding 50 seconds.

### Simulations of the reaction-diffusion model

Simulations were performed with custom code written in MATLAB. Simulations were carried out on a rectangular grid 2×4 mm in physical size, corresponding to 360×720 simulated grid elements with a grid spacing of 0.25 natural length scale units. In all instances, the simulation area was subject to zero flux boundary conditions and the EPS concentration was initially set to zero everywhere. The initial cellular mass was spread in a uniform random distribution over a region covering the bottom 20% of the simulation area. The cellular and EPS concentrations were calculated using a forward Euler method with a spatial and temporal resolution high enough for numerical stability. The time step (*dt* = 0.01) was chosen to be small in comparison to the dimensionless time scale of the system set by the EPS production rate, and simulations were carried out for 600,000 s (300 dimensionless time units), or until motile groups reached the end of the physical simulation area. To compute phase diagrams, free parameters were sampled in log-base 2 across a large range to ensure that all possible classes of relevant behaviors were comprehensively explored. For each point in the phase space, three distinct random initial conditions were tested. We performed a total of 570 simulations for Fig. 6A and 504 simulations for Fig. 9B, varying initial biomass and bias force over the ranges shown.

### Parameter estimation

The EPS production rate *k_s_* and the maximum cellular mobility *m*_0_ set the fundamental length and time scales of the biophysical model. Both of these parameters were estimated from time-lapse imaging data. We assume that the optical halo around groups of cells is formed by the liquid (EPS)/air interface under which the cells reside. For regions where the width of the halo was small compared with the size of the EPS covered region, we assumed that the movement of this interface resulted from production of new EPS, and thus used this edge to approximate the area covered by EPS. We estimated the EPS production rate at 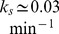 by observing the rate of increase of this area over time, and by assuming that the EPS was approximately as thick as the 1-µm thick monolayer of cells from which it was secreted. We estimated the maximum cellular mobility at 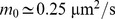 by measuring the root-mean-squared displacements of single cells over time in the absence of light and in a region where EPS had clearly been deposited, thereby assuring that cells could move relatively freely and in unbiased directions.

### Morphological analysis of simulation data

Custom MATLAB software was written to quantify the morphological features of the simulations. Simulations in which less than 50% of the cell mass had moved into fresh territory by the end of the simulation were considered non-motile (black dots in phase diagrams). For the majority subset that was motile, we used an adaptive threshold to determine the position of the moving cellular front as a function of time. We calculated the standard deviation of the points forming the cell front normalized by the mean distance that the cell front had moved, yielding a dimensionless metric of the growth rate of the morphological instability; a perfectly flat cell front would have a value of zero, and a highly unstable front with finger-like projections would have a value close to 1. Simulations that showed an instability growth rate below 0.01 were considered to have a stable front without distinct groups (gray dots in phase diagrams). Simulations with front instability >0.01 exhibited distinct cellular groups that moved toward the simulated light source. In Fig. 6A these points were colored according to their bias force, while in Fig. 9B these points were colored red since the bias force was fixed. For simulations without EPS decay (Figs. 3–7), the distinct motile groups (if present) were segmented to quantify their biomass (measured as an integral from the beginning of the EPS trail), speed (measured from the group center-of-mass), and ramp time.
